# Optimal knee flexion angle during medial patellofemoral ligament reconstruction graft tensioning in a cadaveric model

**DOI:** 10.1002/jeo2.70450

**Published:** 2025-10-13

**Authors:** Robert C. Gillis, Brent G. Parks, Daryl C. Osbahr

**Affiliations:** ^1^ Department of Orthpedic Surgery WellSpan Orthopedics, WellSpan Gettysburg Hospital Gettysburg Pennsylvania USA; ^2^ Department of Orthpedic Surgery MedStar Orthopaedic Institute, MedStar Union Memorial Hospital Baltimore Maryland USA; ^3^ Department of Orthpedic Surgery Rothman Orthopaedic Institute, Rothman Orthopaedics Florida Orlando Florida USA

**Keywords:** biomechanics, medial patellofemoral ligament, MPFL, patella dislocation, patella instability

## Abstract

**Purpose:**

To determine the optimal knee flexion angle for graft tensioning of the MPFL reconstruction to minimize patellofemoral contact pressures.

**Methods:**

Ten cadaveric knees underwent MPFL reconstruction using a hamstring auto‐graft. The graft was fixed to the patella at 40% of length from the proximal tip, secured with suture tied over a bone bridge. On the femoral side, the graft was placed at the anatomic footprint and was draped over a modified pedicle screw with a soft tissue washer. The graft was tensioned at 0°, 15°, 30°, 45° and 60° of knee flexion with 300 N of force. Patellofemoral joint contact pressures were measured at 30°, 45°, 60°, 75° and 90° of knee flexion. Specimens were tested in intact MPFL and reconstructed state. Collinear compressive forces were applied using a custom jig on a load frame. Contact pressure measurements were recorded using the I‐scan system. Data were analyzed comparing different angles of tensioning in the intact, cut MPFL, and reconstructed states at different angles of knee flexion Data were analyzed with repeated measures analysis of variance (ANOVA) with *p* < 0.05 considered significant.

**Results:**

No statistical differences were found in patellofemoral contact pressures when comparing different angles of tensioning in the intact MPFL, or reconstructed state at different angles of knee flexion.

**Conclusion:**

At all angles of knee flexion tested, no significant differences were found in the patellofemoral contact pressures. Cadaveric studies have multiple limitations and most important in this study is the older age of the specimens (average age of 83 years in this study) as most patella instability patients are on the younger side. This study suggests that there is no ideal angle of knee flexion at which to tension an MPFL reconstruction and thus no specific knee flexion angle which is superior to another angle when tensioning the MPFL in a cadaveric model.

**Study Design:**

Controlled laboratory study.

**Level of Evidence:**

N/A.

**Abbreviations**
ANOVA: analysis of variance
MPFL: medial patellofemoral ligament
TT‐TG: tibial tubercle–trochlear groove

## INTRODUCTION

The incidence of patella dislocations is 5.8 per 100,000 and the recurrence rate is from 15% to 44% with nonoperative treatment. The medial patellofemoral ligament (MPFL) is the primary stabilizer of the patellofemoral joint preventing lateral dislocations [2, 5, 7]. Reconstruction of the MPFL is a procedure used in the treatment of patella dislocation [17].

Multiple studies evaluated various components of MPFL reconstruction, particularly as it applies to contact pressures of the patellofemoral joint. Huberti et al. [9] in 1984 was one of the first examine patellofemoral contact pressures. They studied the pressures as they related to the Q‐angle of the knee. Beck et al. [3] in 2007 studied contact pressures and lateral patella translation after MPFL. Most recently, Melegari et al. [13] evaluated contact pressures after MPFL isometric and nonisometric reconstructions. While these studies are important, they do not address the patellofemoral contact pressures regarding the knee flexion angle at the time of MPFL tensioning.

The MPFL provides 50%–60% of lateral restraint for 0°–30° of knee flexion [4]. Graft tensioning is of critical importance during MPFL reconstruction, and the patella should be in the trochlear groove during the first 30 degrees of flexion [7, 20]. Additionally, a properly tensioned reconstructed MPFL should allow 2–3 patella quadrants of translation [16].

Tensioning of the ligament at the time of reconstruction is important. Any time a ligament is reconstructed there is the risk of overtightening or under tightening. Over‐tightening the MPFL may lead to excessive forces on the patellofemoral joint, thus increasing contact pressures, resulting in a painful joint, restricted range of motion, and early arthritis [19, 21]. Over‐tightening the graft can be the result of location of femoral fixation, tension of graft at the time of fixation, and/or the degree of knee flexion at the time of fixation [21]. Under‐tightening may lead continued patella instability.

The *JAAOS* review article, ‘Guidelines for Medial Patellofemoral Ligament Reconstruction in Chronic Lateral Patellar Instability’ concluded that most authors now state that the MPFL is nonisometric over the complete range of knee motion [16]. In separate cadaveric knee models, both Steensen et al. [17] and Victor et al. [22] concluded that the MPFL is nonisometric. It was found that the proximal bundle of the MPFL was tauter at zero, whereas the distal bundle was tauter at 30° of knee flexion [22]. This is in contrast to Stephen et al. [18] who demonstrated in a cadaveric study that the native MPFL is almost isometric through zero to 110° of knee flexion.

The angle of knee flexion during the MPFL reconstruction is surgeon‐dependent. The literature does not specify a recommended knee flexion angle during MPFL reconstruction. The most comprehensive study, ‘Knee flexion angle during graft fixation for MPFL reconstruction: A systematic review of outcomes and complication’ by Patel et al. [15], was published in *Arthroscopy* in 2019. They searched three databases (PubMed, EMBASE and MEDLINE) from database inception to January 2018. The studies were grouped based on flexion angle used during graft fixation: low (0°–30°) and high (45°–90°) flexion angle group. Seventeen studies of 3399 met their inclusion criteria. They concluded that the knee flexion angle during MPFL graft fixation ranged from 20° to 90°. Graft fixation at low and high knee flexion angles during MPFL reconstruction showed excellent patient‐reported outcomes and low patellar re‐dislocation rates overall, with no clear differences between the two groups based on the currently available data [15]. Multiple studies have evaluated contact pressures after MPFL with regards to different parameters as reviewed above [3, 9, 13] To date, no study has evaluated the ideal angle of knee flexion during the tensioning and fixation of the MPFL during reconstruction, particularly as it pertains to contact pressure in the patellofemoral joint. The angle of knee flexion may be a critical portion of the surgical reconstruction to optimize patient outcomes. Thus, the objective of this study is to determine the optimal knee flexion angle of MPFL reconstruction in a cadaveric specimen. The hypothesis is that an ideal knee flexion angle exists at which to tension the MPFL during reconstruction that will minimize contact pressures of the patella on the femoral trochlea in the cadaveric knee.

## METHODS

This is a controlled cadaveric study design using custom MTS jig to test patellofemoral contact pressures over different knee angles before and after MPLF reconstruction at selected angles of knee flexion during reconstruction. Ethics committee approval was not required.

Ten fresh‐frozen cadaveric knees, from the mid‐thigh through mid‐leg, were thawed at room temperature for 24 h and used in this study. All the specimens had a disrupted extensor mechanism as the specimens were transected mid‐femur. Specimens had an average age of 83.1 (range, 68–96) years, three females and seven males. Specimens from four matched pairs and two non‐matched specimens were used. All specimens underwent all conditions during testing and were not randomized into groups.

**Figure 1:**
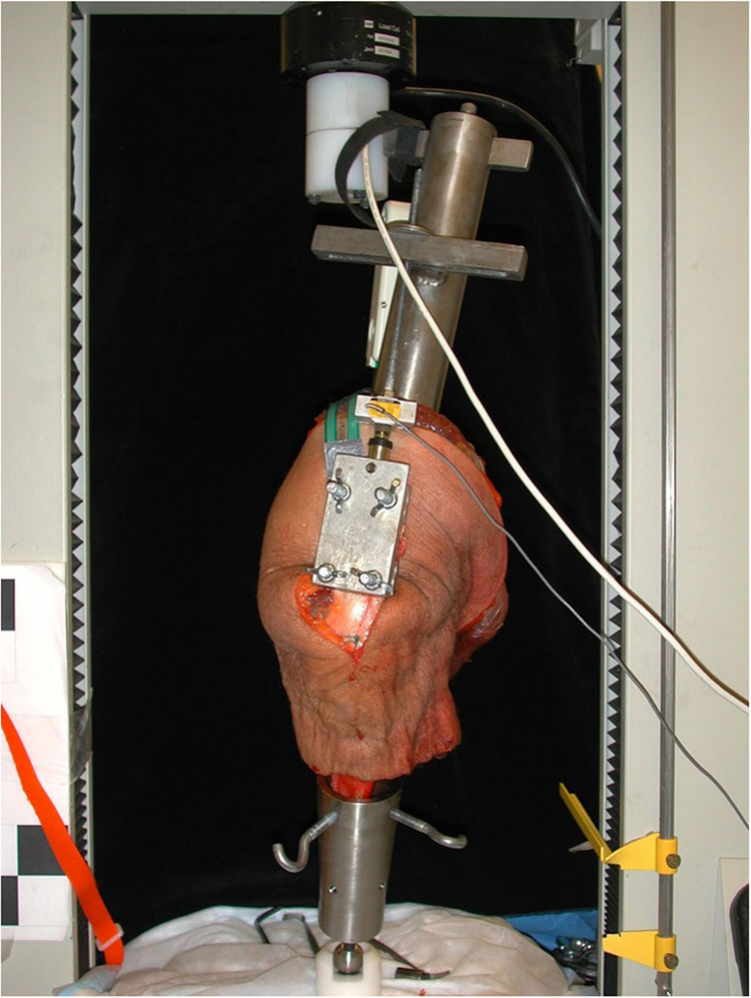
Cadaveric knee attached to custom MTS jig.

Testing was conducted on a custom jig as previously described [13] (Figure 1).

**Figure 2:**
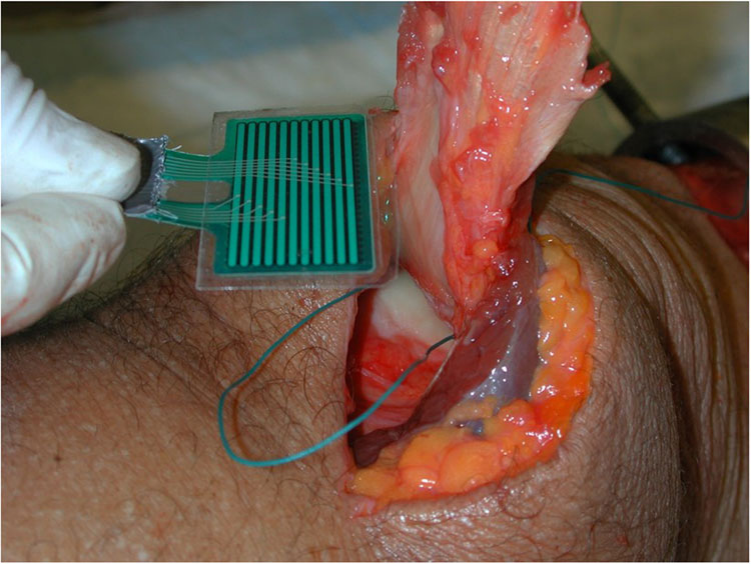
Placement of the I‐Scan sensor in the patellofemoral joint of cadaveric knee.

Contact areas and pressures were measured in the same lab as previously described by Melegari et al. [13] Patellofemoral contact pressure was measured using the I‐Scan system (Tekscan Inc.), a force and pressure measurement system used to determine pressure distributions in joints. The I‐Scan system uses a paper‐thin, high‐resolution sensor placed between articulating surfaces. Sensel density of the sensor was 27.6 sensels/cm^2^, and the size of the sensor was 48 × 23 mm [3]. The sensor was inserted through the superior synovial pouch and centered under the patella (Figure 2).

The sensor was then stitched proximally and distally to the extensor mechanism with one suture of 2.0 Ethibond (Ethicon Inc.). This secured the sensor in the centered position under the patella and allowed gliding of the sensor with flexion and extension of the knee. Sensors were calibrated before use in accordance with the manufacturer's guidelines, and results were interpreted using Tekscan software [3, 13].

**Figure 3:**
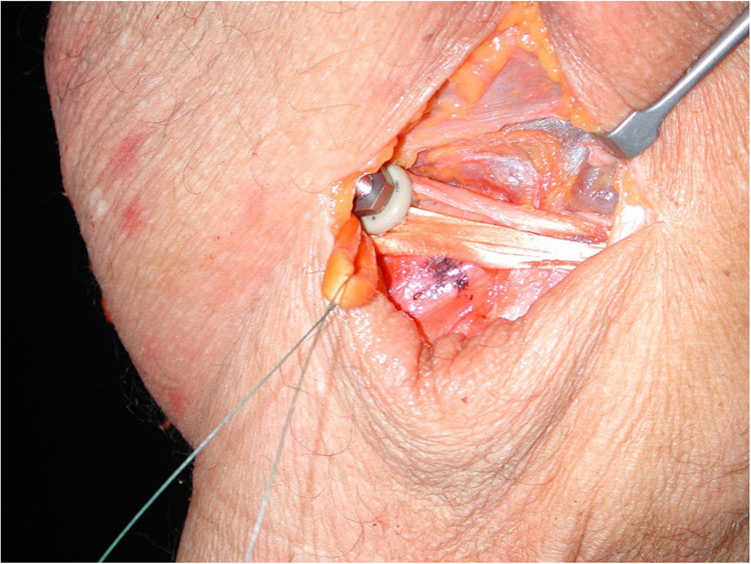
Graft secured to the femur with a modified pedicle screw and a soft tissue washer on a cadaveric knee.

Each specimen was initially pressure tested in the intact MPFL state. Pressure was measured at knee flexion angles of 30°, 45°, 60°, 75° and 90°. The MPFL was then transected, and loading was repeated at the same knee flexion angles. The semitendinosus and gracilis tendon were then harvested in the normal fashion with a tendon stripper. The graft was fixed to the patella at 40% of length from the proximal tip and secured with #1 nonabsorbable suture through bone tunnels and tied over a bone bridge via an MPFL docking technique [1, 23]. On the femoral side, the graft was placed at the midpoint between the medial femoral epicondyle and the adductor tubercle and 10 mm inferior to the adductor tubercle [23]. The graft was secured to the femur via a modified pedicle screw and a soft tissue washer (Figure 3).

The graft was tensioned with 300 N of force, as previously described by Melegari et al. [13], and fixed to the femoral side with a soft tissue washer. Initially, the graft was tensioned at 0° of knee flexion and patellofemoral joint contact pressure was measured at 30°, 45°, 60°, 75° and 90° of knee flexion. The graft was then released on the femoral side, re‐tensioned at 15°, and retested at the same degrees of knee flexion. This process was repeated with graft tensioning at 30°, 45° and 60° of knee flexion.

Power analysis based on a previous studyfound that 10 specimens in each group would give 80% power to detect a 10% change in contact pressure at the 0.05% probability level [13]. Repeated measures analysis of variance (ANOVA) was used to compare pressure data using SigmaStat 3.5 (Systat Software, Inc.) with a *p* < 0.05 considered significant.

## RESULTS

No significant differences in patellofemoral contact pressure were found when comparing different angles of tensioning in the intact MPFL, and MPFL reconstructed states at different angles of knee flexion (Table 1). The highest pressure in an MPFL reconstruction was found at a knee flexion angle of 45° and an MPFL tensioning knee angle of 60° (245 kPa). The lowest pressure in an MPFL reconstruction was found at a knee flexion angle of 30° and an MPFL tensioning knee angle of 60° (205 kPa). The lowest pressure was found with the intact MPFL at 30° of knee flexion (193 kPa) and the highest seen at 60° of knee flexion (227 kPa).

**Table 1:** Mean patellofemoral pressure kPa at measured knee flexion angle.

Pressure (kPa) mean ± SD at knee angle							
		MPFL tensioning knee angle					
Knee angle	Intact MPFL	0°	15°	30°	45°	60°	*p* value
30°	192.6 ± 98.9	210.5 ± 124.5	209.0 ± 112.1	213.6 ± 128.2	223.0 ± 112.9	205.5 ± 123.4	0.55
45°	222.0 ± 101.3	225.4 ± 99.4	232.5 ± 96.9	236.9 ± 95.1	240 ± 106.6	245.3 ± 110.0	0.06
60°	227.8 ± 94.4	233.1 ± 92.0	234.8 ± 94.0	229.7 ± 96.5	227.7 ± 91.6	231.6 ± 83.2	0.95
75°	209.2 ± 90.0	221.7 ± 77.7	213.7 ± 76.4	213.7 ± 79.1	212.3 ± 78.3	211.7 ± 74.4	0.57
90°	226.5 ± 84.8	230.1 ± 83.0	228.6 ± 93.9	222.8 ± 97.5	223.8 ± 92.8	223.1 ± 77.6	0.93

## DISCUSSION

This study evaluated if there is an optimal knee flexion angle at which to tension the MPFL during reconstruction with regard to patellofemoral contact pressures post‐MPFL reconstruction. In this cadaveric model, at all angles of knee flexion tested, no significant differences were found in the patellofemoral contact pressures. This suggests no ideal angle of knee flexion exits at which to tension an MPFL reconstruction in a cadaveric model. Care must be taken not to directly extrapolate these results to healthy patients as this was a cadaveric study which presents limitation to extrapolation.

Lateral patella dislocations can be devastating to any athlete, from recreational to elite. Appropriate tensioning of the MPFL during reconstruction is critical to good outcomes in lateral patella dislocations because reconstruction of this ligament is one of the major stabilizers preventing recurrent dislocation.

The MPFL is the primary stabilizer of the patellofemoral joint preventing lateral dislocations [5]. The other major stabilizers to lateral dislocation are the medial patellotiobial ligaments and the vastus medialis obliquus [1]. Ninety‐nine percent of the time, the patella will dislocate laterally and the MPFL is injured roughly 87% of the time [10, 16] The MPFL provides roughly 55% of the medial constraint and is therefore one of the major passive check reigns to dislocation [6, 11, 12, 14]. The recurrence rate after a first dislocation ranges 15%–44% with nonoperative treatment [10, 12].

In patients with recurrent dislocations, MPFL reconstruction is one of the preferred surgical treatments. Multiple issues can predispose a patient to lateral patella dislocations including trochlear dysplasia, patella alta, a tibial tubercle—trochlear groove (TT‐TG) distance greater than 2 cm, inadequate vastus medialis obliquus strength, excessive femoral anteversion, external tibial rotation, and lower extremity malalignment [1, 8, 11].

Contact between the patella and the trochlear grove begin with the distal aspect of the patella at roughly 20° of knee flexion and continue through the arc to the proximal pole of the patella at roughly 90° of knee flexion [11]. This is consistent with our data that shows a trend (*p *= 0.06) though not significant for 45° of knee flexion in regard to patellofemoral contact pressure. Graft tensioning is of critical importance during MPFL reconstruction, and the patella should be in the trochlear groove during the first 30° of flexion [20]. Additionally, a properly tensioned reconstructed MPFL should allow two to three patella quadrants of translation [23]. Ideally, the patella translation after MPFL reconstruction should match the uninjured contralateral limb [16, 19]. The concept that MPFL tensioning should be done with knee at a minimum of 30° flexion to ensure the patella is located within the trochlea groove can be called into question with the current study. This study demonstrated that there is no difference in patellofemoral contact pressures for MPFL tensioning at 0° and 15° compared to the more traditional angle of greater than 30° to ensure patella location in the groove.

Overtightening is one of the potential threats to the stability of the reconstructed MPFL and may lead to excessive forces on the patellofemoral joint resulting in a painful joint, restricted range of motion, and early arthritis [21]. These complications can be catastrophic, particularly patellofemoral arthritis. Failure of the MPFL and recurrent instability are still an issue, but they do not require life‐changing arthroplasty to rectify. Overtightening the graft can be the result of the femoral fixation location, tension of graft at the time of fixation, and/or the degree of knee flexion at the time of fixation [21]. The MPFL is particularly important in early flexion for the maintenance of patellar stability and normal kinematics [23]. The question of whether an optimal knee flexion angle exists at which to tension the MPFL had not been addressed prior to this study. This study demonstrates that the knee flexion angle during MPFL tensioning has no significant difference on intra‐articular contact pressures in the cadaveric model.

The data suggests that there is no optimal knee angle for tensioning the graft in MPFL reconstruction in a cadaveric model. No significant differences were found in patellofemoral contact pressure among all of the knee tensioning angles used. Several studies have evaluated various components of MPFL reconstruction, particularly as it applies to contact pressures of the patellofemoral joint. Huberti et al. [9] reported that the highest patellofemoral contact pressures were found with knee flexed to 90° in the intact specimen. This contrasts to this study where no significant differences were found between the flexion angles in both the intact knee as well as the reconstructed knees. This study did, however, find highest pressure in an MPFL reconstruction was found at a knee flexion angle of 45° and an MPFL tension knee angle of 60°.

Beck et al. [3] found that low tensions applied to the MPFL reconstruction stabilized the patella and did not increase medial patellofemoral pressures. In the current study, a low graft tension was used which also did not find any difference between the reconstruction groups. Beck also found that increasing graft tension to higher loads increased contact pressures. This study, however, did not measure pressures over increasing graft tension.

Melegari et al. [13] evaluated contact pressures after MPFL isometric and nonisometric reconstructions. They found no difference in the percentage of medial contact area or pressure between the isometric and nonisometric MPFL reconstructions. Although nonisometric reconstruction was not tested, this study, as did Melegari et al. [13], found no differences between the groups of isometric reconstruction.

Parikh et al. [14] recommend tensioning the MPFL reconstruction while the knee is in roughly 45° of flexion. At this degree, the patella is constrained by the trochlea and can help ensure that the graft is not over tensioned [14, 19]. The MPFL should be isometric from full extension to 70° of flexion. Recently, Patel at al. [15], concluded from a systematic review that knee flexion angles from 20° to 90° during MPFL reconstruction showed excellent patient reported outcomes and low re‐dislocation rates. This study found that tensioning the graft, from full extension to 60° of knee flexion, during MPFL reconstruction there was no difference in patella contact pressures. One cannot rule out the possibility that the graft was over‐tensioned and nonisometric, as this was not evaluated in the study. This study found that tensioning the graft, from full extension to 60° of knee flexion, during MPFL reconstruction demonstrated no difference in patella contact pressures and allows for error either with too little or too much knee flexion during MPFL tensioning. One cannot rule out the possibility that the graft was over‐tensioned and nonisometric, as this was not evaluated in the study.

This study does have several weaknesses. As a cadaveric study, physiological realism is reduced due to a multitude of issues including older age of the specimens (average age of 83 years in this study) as most patella instability patients are on the younger side; a lack of trochlear dysplasia in specimens when many patients who require MPFL reconstruction may also have some degree of trochlear dysplasia; and increased laxity of cadaveric specimens compared to their living counterparts. This is a time zero analysis and there is no potential for healing. All the specimens had a disrupted extensor mechanism as the specimens were transected mid‐femur. There is the concern that the loss of proximal patellar tension may make the patella too loose for reliable conclusion. Additionally, the knees were not cycled. Ideally, one cadaveric knee would have been used for each graft tensioning angle. Due to cost restraints, each knee was used for all five graft tensioning angles. Re‐tensioning on the same specimen increases the risk of graft creep which could potentially bias the results. Additionally, this laboratory setup was a non‐weight bearing model. Testing a weight‐bearing situation was not feasible within the cadaveric study set up. Lastly, pressure differences that may occur in a clinical scenario may exist that could not be detected in this study.

Further investigation is warranted to further extrapolate to the clinical realm. One could collect younger specimens to have the age more in line with patellofemoral instability. With these specimens consider cyclic loading. An important change would be to have an intact extensor mechanism which would need to include, at a minimum, a hemi‐pelvis. These issues are very difficult to overcome given cost and time constraints for most projects. The ideal study, would of course, be a randomized controlled study in clinical practice.

In this cadaveric model, at all angles of knee flexion tested, no significant differences were found in the patellofemoral contact pressures. This suggests no ideal angle of knee flexion exists at which to tension an MPFL reconstruction in a cadaveric model. These findings support the null‐hypothesis of our study. The importance of this study is that it found that tensioning the graft, from full extension to 60° of knee flexion, during MPFL reconstruction demonstrated no difference in patella contact pressures and allows for error either with too little or too much knee flexion during MPFL tensioning. These findings may give flexibility during intraoperative decision‐making. However, tension risks still need to be managed despite these findings and more studies are needed to determine if knee flexion angle has any significance on clinical outcomes after MPFL reconstruction.

## AUTHOR CONTRIBUTIONS

**Robert C. Gillis**: Conduct experiment, data analysis, writing, editing. **Brent G. Parks**: Study design, conduct experiment. **Daryl C. Osbahr**: Study design, data analysis, writing, editing.

## CONFLICT OF INTEREST STATEMENT

The authors declare no conflict of interest.

## ETHICS APPROVAL STATEMENT

Ethics approval was not required for this laboratory study.

## Data Availability

The data that support the findings of this study are available from the corresponding author upon reasonable request.
